# Strukturiertes fallorientiertes Lernen: Eine Win-win-Situation für Forschung, Lehre und Behandlung

**DOI:** 10.1007/s00739-020-00655-3

**Published:** 2020-07-02

**Authors:** Sebastian Ertl, Laurenz Stastka, Henriette Löffler-Stastka

**Affiliations:** 1 Klinik für Psychoanalyse und Psychotherapie und Postgraduate Unit, Teaching Center, Medizinische Universität Wien, Währinger Gürtel 18–20, 1090 Wien, Österreich; 2 Klinik für Innere Medizin II, Abteilung für Angiologie, Medizinische Universität Wien, Wien, Österreich

**Keywords:** E‑Learning, Case-based Learning, Embolie, Interdisziplinär, Lehre, eLearning, Case-based Learning, Embolism, Interdisciplinary, Teaching

## Abstract

Ein modernes „case-based“ E‑Learning ist ein exzellentes Hilfsmittel, um Skills und Kommunikation zu trainieren, da „diagnostic reasoning“ als schematisches Problemlösen gesehen wird. Wir präsentieren einen Patientenfall zum Thema Gerinnungsstörung bei chronischem Substanzmissbrauch, bei dem nur durch interdisziplinäres Vorgehen ein adäquater Erfolg erzielt werden kann. Diesbezüglich ist zu erwähnen, dass zu diesem Thema noch wenig Evidenz vorhanden ist und es noch weiterer Forschung bedarf.

**Zusatzmaterial online:**

In der Online-Version dieses Artikels (10.1007/s00739-020-00655-3) finden Sie weiterführende Literatur zum Thema.

## Abstract (en)

Modern case-based eLearning is an excellent tool for skills and communication training, as diagnostic reasoning can be seen as schema-based problem-solving. We report on a patient with coagulation disorder due to chronic substance abuse, in whom reasonable success can only be achieved with an interdisciplinary approach. In this regard, it should be mentioned that there is still little evidence and that more research is needed on this topic.

## Einleitung

Steigende Patientenzahlen, komplexe Patientenfälle, Dokumentationspflicht für jegliche Behandlungsschritte sowie eine gigantische Wissensproduktion, die umgesetzt und angewendet werden soll, stellen angehende Fachärztinnen und Fachärzte vor immer größere Hürden in ihrer Ausbildung. Laut Cochrane-Verzeichnis gibt es bis jetzt eine Million abgeschlossene randomisiert-kontrollierte Studien. Rund 20.000 Studien wurden alleine 2019 publiziert.

Wie kann dieses enorme Wissen adäquat vermittelt und gelehrt werden?

Wie sollte der Wissenstransfer in der Praxis gestaltet werden?

## Fallorientierte Lehre

Zwischen anerkannter Theorie und praktischer Anwendung können Welten liegen. Diese Kluft kann in vielen Bereichen gefunden werden und wird zumeist von jenen, die an vorderster Front arbeiten, als massive Herausforderung gesehen. Um ein modernes postgraduelles Curriculum entwickeln zu können und um den Weiterentwicklungen in der Facharztausbildung gerecht zu werden, müssen Grundlagen des „lifelong learning“ (LLL) sowie aktuelle Forschungsergebnisse aus der Medizindidaktik berücksichtigt werden. Die Integration neuer State-of-the-Art-Behandlungsmöglichkeiten und deren Training in Aus- und Weiterbildung, wie die psychotherapeutische Medizin in der Psychiatrie, ist hier als Beispiel zu nennen.

Knowles et al. publizierten wie eine Vielzahl anderer Autoren verschiedene Aspekte, die essenziell in der postgraduellen Lehre sind. Bereits in der 1980er-Jahren wurden diese von Merriam zu den 3 wichtigen Säulen des „lifelong learning“ zusammengefasst: die Veränderung des Lernens, der Lebensumstände sowie Bewusstseinsveränderung im Erwachsenenalter.

Für Knowles stellt die intrinsische Motivation den wichtigsten Antrieb für Erwachsene dar. Im Lernprozess ist das Zeitmanagement, beeinflusst durch Fristen, Beruf und den Alltag signifikant anders als bei Jugendlichen. Werden die zu lernenden Inhalte als nicht bedeutungsvoll genug angesehen oder spiegeln sie nicht die eigenen Interessen wider, hat dies wiederum Einfluss auf die Motivation der Weiterbildungsstudierenden.

E‑Learning, die perfekte Lösung für jeden?

E‑Learning is a tool, not a teacher

E‑Learning wird häufig als die ideale Lösung für jeden Lerntyp und jeden Inhalt gesehen.

Coffiedl et al. publizierten in ihrer Metaanalyse über 70 verschiedene Parameter, die in der Literatur verwendet wurden, um verschiedene Lerntypen und Lehrmethoden zu beschreiben. Vielen Arbeiten fehlt es jedoch an Evidenz oder validen Messmethoden, um signifikante Ergebnisse zu präsentieren.

## „Blended learning“

Vor 20 Jahren wurde „blended learning“ (BL) von der amerikanischen Gesellschaft für Lehre (American Society for Training and Development) als wichtigste Möglichkeit, um Studierende zu unterrichten, bezeichnet. Ross & Gage beschrieben BL als den neuen Goldstandard in der Lehre und Garrison et al. als: „Einfach und leicht zugleich – die neue Kunst zu lehren“.

„Blended learning“ verlangt eine Kombination von Präsenzlehre und E‑Learning. Der Fokus wird jedoch zumeist abhängig von der Institution ausgerichtet und wenig oder gar nicht an die Bedürfnisse der Studierenden oder den zu vermittelnden Inhalt angepasst. Dies führt oftmals trotz guter didaktischer Methoden zu schlechteren Ergebnissen gegenüber dem herkömmlichem Unterricht. Auch lehrte uns die aktuelle Situation der SARS-CoV-2-Krise, möglichst auf Online-Arbeit, -Therapie und -Unterricht umzustellen und dabei sinnvolle Distant-learning-Methoden parat zu haben.

Die Herausforderung des „blended learning“ besteht in einer gut durchdachten Integration und Abstimmung. Der mit höheren Ressourcen verbundene Anteil der Präsenztermine muss mit dem zeitaufwendigeren E‑Learning-Bereich anhand aktueller Guidelines kombiniert werden.

## „Case-based blended-learning“

Für angehende Fachärztinnen und Fachärzte stellt die große Fülle an Informationen zu Beginn ihrer klinischen Karriere eine große Herausforderung dar.

„Case-based blended-learning“ hilft bei der Suche nach essenziellen Befunden

„Case-based blended-learning“ stellt eine gute Möglichkeit dar, den Umgang mit dieser Flut zu erlernen und sich zielgerichtet auf die Suche der essenziellen Befunde zu begeben.

## Fallorientierte Lehre – „clinical reasoning“ und „clinical decision making“

In unserem Kurs verwenden wir das Konzept eines fallbasierten „blended learning“ mit Schwerpunkt auf einem online „distant learning“. Aufgrund der jahrelangen Forschung und Entwicklungsarbeit sowie unserer international gut vernetzten Arbeitsgruppe, die ihre Forschungsergebnisse bereits auf zahlreichen Kongressen im In- und Ausland präsentieren durfte, konnten wir uns relativ rasch auf die aktuellen Gegebenheiten anpassen. Damit wurden wir den Gegebenheiten aktuell schnell gerecht, da vorbereitet und seit Längerem international vernetzt.

## Struktur

Inhaltlich werden interdisziplinäre Fälle sowie Fälle zu den folgenden Fachbereichen angeboten:

Psychiatrie (inkl. Psychotherapie und Gesprächsführung), Innere Medizin, Dermatologie, Traumatologie (mit Unfallchirurgie und Orthopädie), Notfallmedizin (interdisziplinäre Fälle mit Unfallchirurgie und Anästhesie), Pädiatrie (mit Schwerpunkt auf Notfällen und Akutversorgung in der Klinik) und Allgemeinmedizin (mit stark interdisziplinärem Charakter).

Der Kurs ist in zwei Teile aufgeilt, im ersten Part müssen Patientenfälle aus den enthaltenen Bereichen gelöst werden. Es werden unter anderem folgende Fachbereiche abgedeckt: Psychiatrie, Psychotherapie, Neurologie, Anästhesie und Intensivmedizin, Innere Medizin und chirurgische Fächer etc. Jeder Fall ist als ein Szenario angelegt, um eine virtuelle Situation in einem Krankenhaus zu simulieren. Interaktive Fragen im Multiple-Choice Format führen zur Lösung eines Falles. Im zweiten Part müssen die Teilnehmerinnen und Teilnehmer selbst einen eigenen Patientenfall anhand ausgewählter Kriterien erstellen und im Anschluss in einem Peer-Review anonym Fälle von Kolleginnen und Kollegen bewerten. Ziel ist es hier, die Situation eines „Lehrenden“ darzustellen und Einblick in die Perspektive eines Supervidierenden zu ermöglichen.

Der strukturelle Aufbau aller Lehrfälle folgt einem standardisierten Schema. Dies wird anhand unseres angeführten Fallbeispiels, das jedoch stark gekürzt wurde, genauer verdeutlicht.

## Fallbericht: Embolie nach Substanzabusus

Die stationäre Aufnahme der Patientin erfolgt über die ZNA (Zentrale Notfallaufnahme) nach Überweisung vom niedergelassenen Allgemeinmediziner.

### Anamnese

Die Patientin berichtet davon, sich die rechte Hand zwischen einer Türklinke und der Wand eingequetscht zu haben. Der Hausarzt beschreibt eine pulslose A. radialis sowie A. ulnaris, bei einer ausreichend perfundierten und seitengleich warmen Extremität. Zur weiteren Abklärung erfolgt eine Verlegung auf die Abteilung für Angiologie.

### Status

Die Patientin präsentiert sich in reduziertem Allgemeinzustand und adipösem Ernährungszustand.

Als Vorerkrankungen sind eine Hypertonie, Z. n. Magenbypass-OP, Z. n. Tonsillektomie, Z. n. Cholezystektomie sowie Nikotin‑, Alkohol- und Cannabisabusus bekannt.

Bei einem ausführlichen Anamnesegespräch fällt die deutlich gesteigerte Affizierbarkeit im negativen Skalenbereich auf. Die Patientin gibt an, unter Durchschlafstörungen zu leiden, nur mehr wenig Hunger zu verspüren und Essen als Last zu betrachten. Zur Zeit der Aufnahme ist die Patientin arbeitssuchend und kümmert sich 3‑mal wöchentlich um die Mutter. Die Patientin fängt beim Erzählen der sozialen Situation zu weinen an.

### Apparative Diagnostik

Befund der Duplexsonographie: Die rechte Arteria subclavia frei durchgängig mit proximalem Pendelfluss. Der distale Bereich der Arteria subclavia zeigt ein niedriges, prästenotisches Flussprofil. Die Arteria axillaris und die Arteria brachialis sowie die Unterarmarterien sind komplett verschlossen. Es gibt eine kaliberstarke Kollaterale im Bereich der Arteria axillaris.

Zu einer weiteren Abklärung wird eine MRT-Untersuchung durchgeführt: Die rechte Arteria subclavia ist regulär kontrastiert und unauffällig. Auch die rechte Arteria axillaris gelangt regulär kontrastiert zur Darstellung. Es findet sich ein langstreckiger Verschluss der Arteria brachialis ab deren Abgang. Dabei zeigt sich ein kräftig kontrastiertes Kollateralgefäß am Oberarm. Am distalen Oberarm rekonstruiert sich die Arteria brachialis kurzstreckig. Die Brachialisgabel lässt sich nicht suffizient kontrastiert darstellen.

Bei einer im Rahmen der MR-Angiographie durchgeführten Bildgebung des Oberbauches zeigt sich ebenfalls eine kurzstreckige KM-Aussparung sowie ein fehlendes „flow void“ der Pfortader, was auf eine Pfortaderthrombose schließen lässt.

### Therapie

- Antikoagulatorische Therapie mit Enoxaparin-Natrium 100 MG 1‑0‑1 und Prostaglandin E1
- Gerinnungskonsil durch die Abteilung der Labormedizin
- Fachärztliches Konsil durch die Abteilung der Psychiatrie
- Gerinnungskonsil: Es ergibt sich kein Hinweis auf eine angeborene oder erworbene Gerinnungsstörung, die ursächlich für die Verschlüsse sein könnte.
- Psychiatrisches Konsil: Mittelgradig depressive Episode sowie psychische und Verhaltensstörungen durch Alkohol und andere Substanzen.
- Weiteres Prozedere: Etablierung einer antidepressiven Therapie mit Trazodon-Hydrochlorid mit Beginn 150 MG 0‑0‑1 und Steigerung alle 2 Tage bis zu einer Zieldosis von 300 mg.
- Bei zunehmend subjektiven Entzugsbeschwerden Beginn von Oxazepam 50 mg ½‑½-0 und Ausschleichen über mindestens 2 Wochen bei Sistieren der Entzugssymptome. Anbindung an eine/n Facharzt/Fachärztin für Psychiatrie und Psychotherapeutische Medizin, bereits im Rahmen des stationären Aufenthaltes die Patientin dahingehend motivieren.

## Empfohlene Maßnahmen

- Strikte Nikotinkarenz, Vorstellung beim niedergelassenen FA für Psychiatrie und Psychotherapeutische Medizin zur weiteren Anbindung und Betreuung bei mittelgradiger depressiver Episode und Substanzabusus
- INR-Kontrolle in der angiologischen Ambulanz nach 6 Wochen

## Zusammenfassung

Der Aufnahmegrund war ein Oberarmarterienverschluss rechts bei Status post Trauma an der rechten Hand ca. 11 Tage davor. Eine MR-Angiografie der betroffenen Gefäßabschnitte zeigte einen Verschluss der Arteria brachialis rechts. Es erfolgte die Etablierung eines blutverdünnenden sowie eines gefäßerweiternden Medikaments. In den sonographischen Kontrollen zeigte sich im Verlauf des stationären Aufenthaltes eine deutlich verbesserte Symptomatik. Als kardiovaskuläre Risikofaktoren ließen sich ein Nikotinabusus seit dem 15. Lebensjahr sowie ein Cannabiskonsum eruieren. Zum Zeitpunkt der Entlassung wurde eine antikoagulatorische Therapie mit Phenprocoumon etabliert. Ursächlich für den Verschluss wird bei Entlassung der anhaltende Cannabiskonsum angesehen.

## Offene Testfragen zum Lehrfall

- Warum wurde zur medikamentösen Therapie Trazodon gegenüber einem SSRI verwendet? (Bitte diskutieren Sie folgende Publikation zur Wirkung von thrombozytärem Serotonin auf die arterielle und venöse Thrombose in Ihrer Überlegung)
- Nehmen Sie Stellung zur psychischen Situation der Patientin (Substanzabusus, Persönlichkeitsfaktoren, soziale Parameter). Wie sehen Sie die Magenbypassoperation?
- Sprechen Sie destruktive Anteile aktiv an? Wenn ja, wie?
- Wie kann der Enttäuschungsprotest der Patientin interpretiert und eine ausreichend gute stabile therapeutische Beziehung etabliert werden?
- Wie kann bei der Patientin eine optimale Behandlungsadhärenz erzielt werden?
- Welche Rolle spielen Protein S und Protein C? Welche Studienergebnisse kennen Sie diesbezüglich?
- Wie gestalten Sie Ihren Kontakt zum Internisten bzw. im interdisziplinären Team?

## Conclusio

Ein modernes „case-based“ E‑Learning ist ein exzellentes Hilfsmittel, um Skills und Kommunikation zu trainieren, da „diagnostic reasoning“ als schematisches Problemlösen gesehen wird.

Kopp et al. postulierten, dass Lehren mit Beispielen nachhaltigeres Wissen vermittelt als die Präsentation von abstrakten Informationen. Dies ist auf einen unterschiedlichen kognitiven Arbeitsanspruch zurückzuführen. Der pädagogische Nutzen von Fehlern sollte außerdem in der Planung von Lehrinhalten berücksichtigt werden, konstantes Feedback zu richtigen oder falschen Schritten bewegt Teilnehmerinnen und Teilnehmer zu einer intensiveren Auseinandersetzung mit den angebotenen Inhalten und festigt langfristiger das neu erlernte Wissen. Ebenso entwickeln sich anhand diffiziler Fälle oftmals neue Forschungsfragen.

## COVID-19

Aufgrund der weltweiten COVID–19-Pandemie mussten viele Universitäten und Akademien ihre Face-to-Face-Kurse aussetzen. Viele Einrichtungen versuchten daher in relativ kurzer Zeit ein „distant learning“ umzusetzen. Unter diesen Umständen wurde unser Kurs von bis zu 1000 Medizinstudierenden gleichzeitig besucht und über 7000 Kommentare verfasst.

## Fazit für die Praxis

- „Case-based blended-learning“ stellt eine gute Möglichkeit dar, um den Umgang mit der Informationsflut im Stationsalltag zu erlernen und sich zielgerichtet auf die Suche der essenziellen Befunde zu begeben.
- E‑Learning wird oftmals ohne evidenzbasierte Forschungsdaten zur Didaktik geplant und umgesetzt.
- Der pädagogische Nutzen von interaktiven Fragen sollte in der Präsentation jeder Kasuistik berücksichtigt werden.
- Komplexe Patientenfälle erfordern ein interdisziplinäres Vorgehen.

## Caption Electronic Supplementary Material





