# Supplementary material for: Structured Case-Oriented Learning: A Win-Win Situation for Research, Teaching and Treatment
Source: psychopraxis. neuropraxis. 2020 Jul 2;23(5):240–5. [Article in German] doi: 10.1007/s00739-020-00655-3 (PMC7330881; doi:10.1007/s00739-020-00655-3)
Supplement: Supplementary file 1 [file 739_2020_655_MOESM1_ESM.docx]

## **Literatur**

1. Tripp D. Critical incidents in teaching (classic edition): Developing professional judgement. Routledge; 2011.

2. Löffler-Stastka H, Fink B, Franz J, Lenz G, Matuszak-Luss K, Sachs G, Tölk A, Wagner E, Aigner M. „Basiscurriculum in psychotherapeutischer Medizin“: Psychotherapeutische Ausbildung in der Psychiatrie – ein Beginn. Psychiatrie und Psychotherapie 2011;7:20-26. DOI:10.1007/s11326-011-0147-8

3. Cross KP. Adults as Learners. Increasing Participation and Facilitating Learning. 1981;

4. Knox AB. Proficiency theory of adult learning. Contemp Educ Psychol. 1980;5(4):378–404.

5. McClusky HY. An approach to a differential psychology of the adult potential. 1970.

6. Mezirow J. A critical theory of adult learning and education. Adult Educ. 1981;32(1):3–24.

7. Freire P. Pedagogy of the Oppressed (Herder and Herder, New York). New York: Herder & Herder; 1970.

8. Merriam SB, Caffarella RS, Baumgartner LM. Learning in adulthood: A comprehensive guide. 2007;

9. Merriam SB. Adult learning and theory building: A review. Adult Educ Q. 1987;37(4):187–98.

10. Knowles MS. The Modern Practice of Adult Education: From Pedagogy to Andragogy: Revised and Updates. Adult Education; 1980.

11. Swanwick T. Understanding Medical Education Second edition. 2014.

12. Papert S. The children’s machine: Rethinking school in the age of the computer. ERIC; 1993.

13. Coffield F, Moseley D, Hall E, Ecclestone K. Learning styles and pedagogy in post-16 learning: a systematic and critical review | VOCEDplus, the international tertiary education and research database [Internet]. 2004 [cited 2020 Mar 27]. Available from: https://www.voced.edu.au/content/ngv%3A13692

14. Rooney JE. Knowledge infusion. Assoc Manage. 2003;55(5):26.

15. Ross B, Gage K. Global perspectives on blending learning. Handb Blended Learn Bonk, JC, Graham, RC, Eds. 2006;155–68.

16. Garrison DR, Kanuka H. Blended learning: Uncovering its transformative potential in higher education.

17. Garrison DR, Archer W. A Transactional Perspective on Teaching and Learning: A Framework for Adult and Higher Education. Advances in Learning and Instruction Series. ERIC; 2000.

18. Sharpe R, Benfield G, Roberts G, Francis R. The undergraduate experience of blended e-learning: a review of UK literature and practice. High Educ Acad. 2006;1–103.

19. Garrison DR, Cleveland-Innes M. Critical factors in student satisfaction and success: Facilitating student role adjustment in online communities of inquiry. In: Invited paper presented to the Sloan Consortium Asynchronous Learning Network Invitational Workshop, Boston, MA. 2003.

20. Williams B. Case based learning - A review of the literature: Is there scope for this educational paradigm in prehospital education? Emerg Med J. 2005;22(8):577–81.

21. Wadowski PP, Litschauer B, Seitz T, Ertl S, Löffler-Stastka H. Case-based blended eLearning scenarios—adequate for competence development or more? Neuropsychiatrie. 2019;33(4).

22. Turk B, Ertl S, Wong G, Wadowski PP, Löffler-Stastka H. Does case-based blended-learning expedite the transfer of declarative knowledge to procedural knowledge in practice? BMC Med Educ. 2019;19(1).

23. Löffler-Stastka H, Seitz T, Billeth S, Pastner B, Preusche I, Seidman C. Significance of gender in the attitude towards doctor-patient communication in medical students and physicians. Wien Klin Wochenschr. 2016;128(17–18):663–8.

24. Seitz T, Gruber B, Preusche I, Loffler-Stastka H. What causes the decrease in empathy among medical students during their university training?/Rückgang von Empathie der Medizinstudierenden im Laufe des Studiums--was ist die Ursache? Z Psychosom Med Psychother. 2017;63(1):20–40.

25. Ertl S, Löffler-Stastka H. Case Based Blended Learning (CBBL) – a strategy to foster the transfer of declarative to procedural knowledge or more? In: AMEE Conference Vienna. 2019.

26. Ertl S, Löffler-Stastka H. Vienna tracking students in 25.000 exam results. In: AMEE Conference Basel. 2018.

27. Loosveld LM, Van Gerven PWM, Vanassche E, Driessen EW. Mentors’ Beliefs About Their Roles in Health Care Education: A Qualitative Study of Mentors’ Personal Interpretative Framework. Acad Med. 2020 Jan;

28. Naber K, Gauchel N, Mammadova-Bach E, Mauler M, Stallmann D, Kröning P, Bode C, Braun A. Dürschmied D. Abstract 1367 Die Wirkung von thrombozytärem Serotonin auf die arterielle und venöse Thrombose [Internet]. [cited 2020 Jun 1]. Available from: https://dgk.org/kongress_programme/jt2019/aV1159.html

29. Koh GCH, Khoo HE, Wong ML, Koh D. The effects of problem-based learning during medical school on physician competency: A systematic review. Cmaj. 2008;178(1):34–41.

30. Berkhof M, van Rijssen HJ, Schellart AJM, Anema JR, van der Beek AJ. Effective training strategies for teaching communication skills to physicians: An overview of systematic reviews. Vol. 84, Patient Education and Counseling. 2011. p. 152–62.

31. Mandin H, Harasym P, Eagle C, Watanabe M. Developing a "clinical presentation" curriculum at the University of Calgary. Acad Med J Assoc Am Med Coll. 1995;70(3):186–93.

32. Mandin H, Jones A, Woloschuk W, Harasym P. Helping students learn to think like experts when solving clinical problems. Acad Med J Assoc Am Med Coll. 1997;72(3):173–9.

33. Kopp V, Stark R, Fischer MR. Fostering diagnostic knowledge through computer-supported, case-based worked examples: Effects of erroneous examples and feedback. Med Educ. 2008;42(8):823–9.

34. Kiesewetter J, Ebersbach R, Görlitz A, Holzer M, Fischer MR, Schmidmaier R. Cognitive problem solving patterns of medical students correlate with success in diagnostic case solutions. PLoS One. 2013;8(8).
